# OLED illuminated metasurfaces for holographic image projection

**DOI:** 10.1038/s41377-025-01912-z

**Published:** 2025-08-27

**Authors:** Junyi Gong, Mohammad Biabanifard, Kou Yoshida, Graham A. Turnbull, Andrea Di Falco, Ifor D. W. Samuel

**Affiliations:** SUPA, School of Physics and Astronomy, University of St Andrews, North Haugh, St Andrews, Fife KY16 9SS UK

**Keywords:** Organic LEDs, Metamaterials

## Abstract

Organic light-emitting diodes (OLEDs) are thin film optoelectronic devices that feature simple fabrication, light weight and broad tunability, which makes them widely used in mobile phone and TV displays. As a flat and surface-emitting light source, OLEDs are also used in emerging applications such as optical wireless communications, biophotonics and sensing, where the ability to integrate with other technologies makes them good candidates to realise miniaturised photonic platforms. Control of the OLED far-field emission is increasingly important for both displays and these emerging applications. At present, however, studies mainly focus on tuning the electroluminescence (EL) spectrum and emission directionality. Fine-tuning of the far-field emission is particularly challenging and is limited by the low spatial coherence of OLEDs. In this work, we show that it is possible for a single OLED to project a high-resolution image when combined with a holographic metasurface as a compact projection system. The metasurface-OLED projector allows us to directly manipulate the OLED far-field emission and display holographic images on a screen. Here, we further show how the projected image quality relates to the spatial coherence length and the spectrum of the OLED. We believe our demonstration provides a path towards a miniaturised and highly integrated metasurface display.

## Introduction

Organic semiconductors are carbon-based materials with wide tunability^1,2^, low cost^3^ and good processability^4,5^. Their outstanding optoelectronic properties have made them very suitable for applications in displays^6^, photovoltaics^7^ and lasing^8^. Their use as organic light-emitting diode (OLED) displays is the most developed application, and they are now found widely in mobile phones and televisions. Beyond display technologies, other OLED applications are emerging in optical wireless communications (OWC)^9^, bioimaging^10^ and sensing^11^.

To address the varied demands of different applications, it is highly advantageous to manipulate the far-field emission properties of OLEDs. Current studies are limited to tuning the electroluminescence spectrum and directionality properties^12,13^. An OLED is an incoherent light source giving a divergent emission profile^14^. Manipulation of the OLED emission to generate highly detailed images is very challenging and remains unexplored.

A potential solution to manipulate OLED emission is to use a holographic metasurface. Holographic metasurfaces are sub-wavelength-scale thin film structures that are capable of modulating incident light^15^. They are widely used in applications such as augmented reality^16^, imaging^17^, sensing^18^, anticounterfeiting and security encryption^19^. However, most reported holographic metasurfaces are designed for coherent light sources (lasers) and are unsuitable for use with incoherent light sources like OLEDs^20^. To date, there are only a few reported metasurfaces using incoherent light sources^21–24^. Most of them are based on polarized and collimated light, which potentially leads to complicated setups and limits further deployment in everyday applications.

**Fig. 1:**
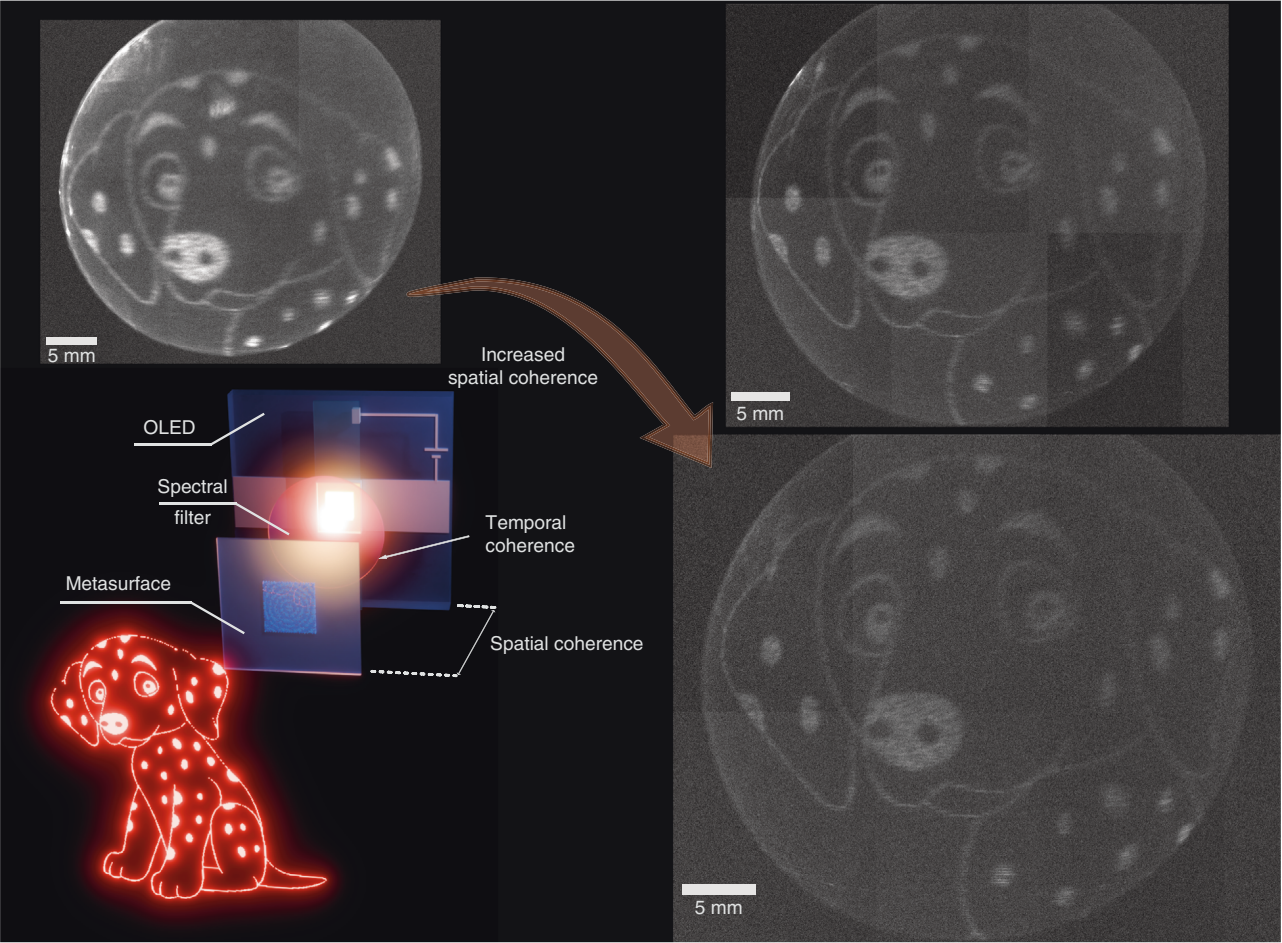
Concept view (bottom left) of the proposed OLED illuminated holographic image projection system, together with three holographic images recorded under OLED illumination with different spatial coherence. The OLED was placed at various distance (top left: 3 cm, top right: 5 cm and bottom right: 6 cm) away from the metasurface

In this paper, we develop a new type of optoelectronic device by combining an OLED and a metasurface. The compact system (shown in Fig. 1) consists of a holographic metasurface, a bandpass filter, and an OLED. The metasurface used in this work was designed for coherent light sources. By manipulating the configuration of the OLED-metasurface system, we enhanced the degree of spatial coherence, allowing the holographic metasurface to directly modulate the unpolarized OLED emission and project a holographic image onto a screen. We further explored the effects of the spatial coherence and FWHM of the OLED electroluminescence spectrum on the holographic image quality. By bringing together the advantages of organic optoelectronics and nanophotonics, we establish a new paradigm for holographic displays.

## Results

**Fig. 2:**
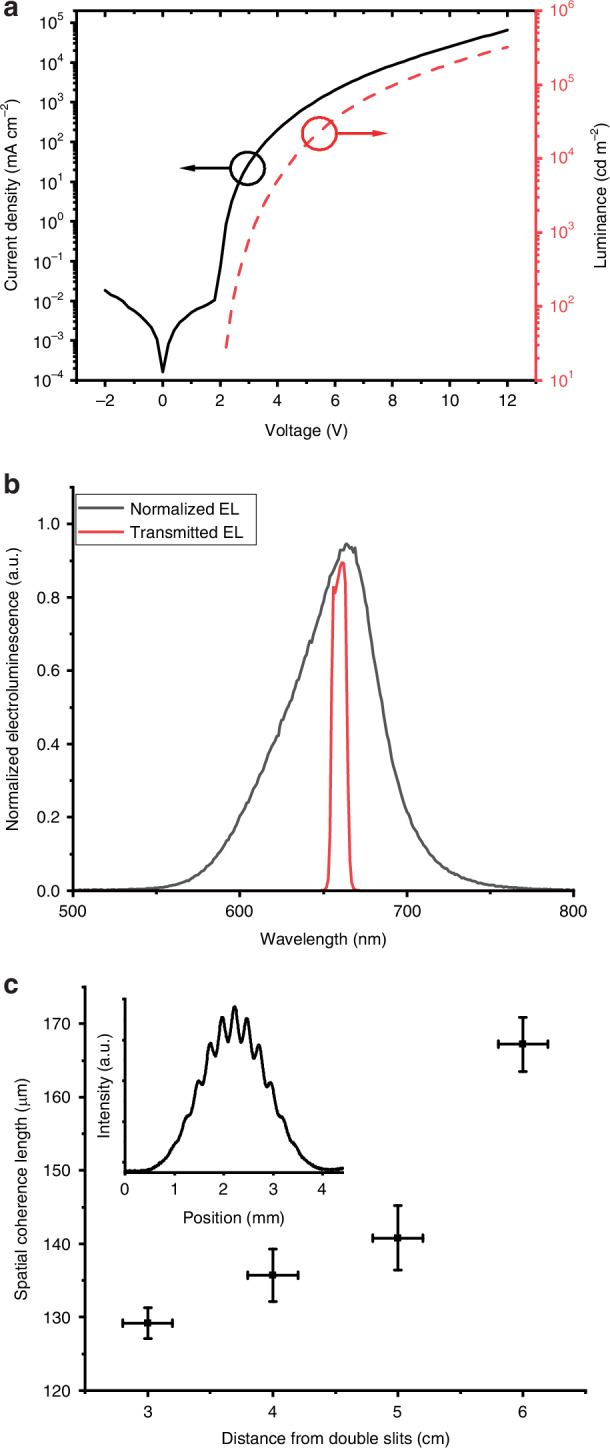
OLED operating characteristics. **a** Current density, and luminance as a function of OLED driving voltage. **b** OLED electroluminescence spectrum with and without bandpass filter. **c** Spatial coherence length as a function of the distance between the OLED and double slits, inset is the measured fringe pattern when OLED is 6 cm away from the double slits, error bars are uncertainties of the measurement

To project an image, a bright and stable OLED is required. We designed an OLED with high brightness^9^ and long operational lifetime^25^. The device stack (Fig. S1) was thermally evaporated on a silicon substrate using a top-emitting configuration. The device consists of a bottom aluminium electrode and a top transparent silver electrode. A fluorescent emitter 4-(dicyanomethylene)-2-tert-butyl-6-(1,1,7,7-tetramethyljulolidin-4-yl-vinyl)-4H-pyran (DCJTB) doped in di(naphthalen-2-yl)perylene (DNP) and tris(8-hydroxy-quinolinato)aluminium (Alq_3_) cohost was sandwiched between charge blocking and charge transport layers (details in Methods). A photograph of the fabricated device is shown in Fig. S2a. The silicon substrate provides good heat dissipation during OLED operation to enable stable operation at high brightness. We use a p-i-n structure with doped hole and electron transporting layers to facilitate charge carrier injection and transport^26^. We characterised the OLED under direct current (DC) operation. As shown in Fig. 2a, our design enables the OLED to operate at up to 64 A/cm^2^ (at 12 V) and a high luminance of 3.2 × 10^5^ cd/m^2^. The electroluminescence peaks at 664 nm with a full width at half maximum (FWHM) of about 63 nm (Fig. 2b).

OLEDs are known to have low spatial coherence^14^, so they are not usually suited for holography. The spatial coherence of an OLED could, however, be enhanced by spatial filtering^27^, microcavity effects or incorporating diffractive optics in the device^14^. It can also be manipulated by extending the distance from the light source to the hologram^28^. We first examined the spatial coherence of our OLED at different distances. To quantify the degree of spatial coherence, we illuminated a pair of parallel slits with an OLED located at different distances and recorded the corresponding interference patterns. The double slits used had a width of 40 µm and a centre-to-centre distance of 250 µm. The spatial coherence length was calculated using a Gaussian–Schell model^14^. Details of the experiment and calculation of the spatial coherence length can be found in the “Methods”. As shown in Fig. 2c, the spatial coherence length is about 130 µm at the slits for an OLED placed 3 cm away from the slits, increasing to about 170 µm at a distance of 6 cm.

To create the holographic image, we designed a phase-only holographic metasurface. Our metasurface works in transmission, and each meta-atom functions as a cylindrical truncated waveguide. Each meta-atom produces a phase change proportional to the effective refractive index of the propagating mode, which can be easily controlled by tuning the diameter of the waveguide. The phase profile of the holographic metasurface was numerically calculated using a customised Gerchberg-Saxton algorithm^29^. This was achieved by iteratively propagating the light between the metasurface plane and the holographic image plane, in this case using a Rayleigh-Sommerfeld propagator. The hologram had 1667 by 1667 pixels of size 300 × 300 nm, and the holographic image had 333 by 333 pixels of 500 × 500 μm.

Subsequently, this phase profile was translated into a distribution of meta-atoms specifically selected to apply the desired phase modulation (see Table S3).

**Fig. 3:**
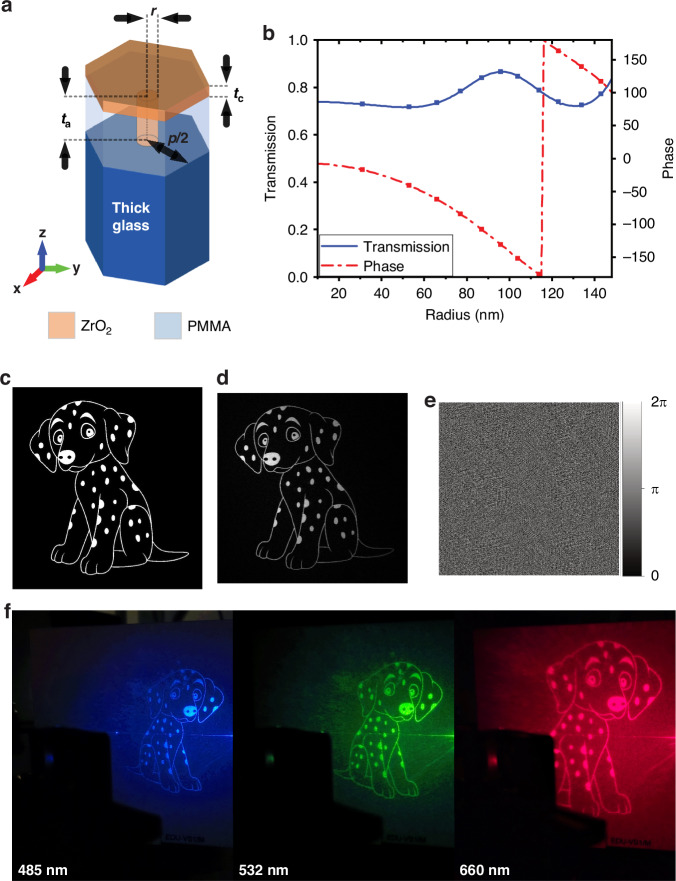
Design and characterisation of the metasurface. **a** Design of the meta-atom where *p* = 300 nm, *t*_a_ = 700 nm, and *t*_c_ = 180 nm. **b** The phase modulation as a function of meta-atom radius. The squares report the discrete phase values chosen for the practical implementation. **c** The target image. **d** The reconstructed holographic image. **e** The designed phase profile. **f** Holographic images measured with coherent light at 485, 532, and 660 nm. The images appear mirrored with respect to the design, as they are projected onto a screen

The meta-atoms are based on transparent zirconium dioxide (ZrO_2_) with the geometry shown in Fig. 3a^30^. The ZrO_2_ pillars are embedded in a 700 nm thick PMMA layer and encapsulated by a thick glass layer at the bottom and a thin ZrO_2_ layer at the top. Our method does not require any liftoff or Reactive Ion Etching (RIE) process, which greatly simplifies the fabrication. A cross-sectional Scanning Electron Microscope (SEM) image of the metasurface is shown in Fig. S2b. We have conducted multiple fabrication runs with a yield of 91% (10 successful samples out of 11 fabrications) and found that the metasurface exhibits significant robustness to fabrication imperfections. Details can be found in supporting information Fig. S3. Figure 3b shows the relationship between the pillar’s diameter and the dephasing for a wavelength of 532 nm. The specifics of the simulation and fabrication are detailed in the methods section. Figure 3c–e present the target image for the hologram, the reconstructed image, and the corresponding phase profile of the designed hologram, respectively. The first-order image was designed to be tilted at 30 degrees from the zeroth-order diffraction. The diffraction efficiency of the metasurface was 27% at 30 degrees (see supporting information). We have summarised focusing efficiency and diffraction efficiency of our work and recent literature reports in the supporting information (Table S1). We note that diffraction efficiency depends on the image, and in any case, we were aiming to demonstrate proof of principle rather than to maximise the efficiency of our metasurface.

We first characterised the fabricated metasurface under laser illumination at different wavelengths. As shown in Fig. 3f, the metasurface projected images of a Dalmatian dog on a viewing screen at 30 degrees to the normal of the metasurface. The fabricated metasurface clearly showed the capability of manipulating the incident light of a highly spatially coherent source. We further characterised the metasurface under illumination of different polarizations, wavelengths and incident angles. Detailed discussion and analysis, including contrast-to-noise ratio (CNR) and signal-to-noise ratio (SNR) of the holographic images, can be found in the supporting information.

**Fig. 4:**
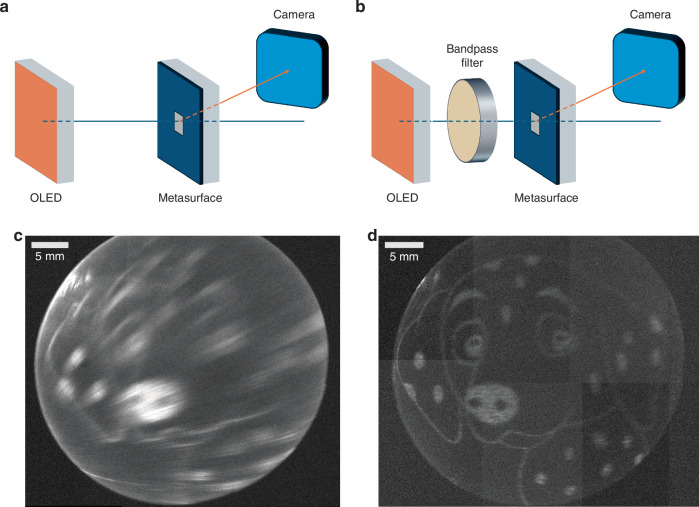
Metasurface under OLED illumination. **a** OLED illuminated metasurface setup without bandpass filter. **b** OLED illuminated metasurface setup with bandpass filter. Recorded holographic image under OLED illumination **c** without bandpass filter, and **d** with bandpass filter

We next characterised the metasurface under OLED illumination. The schematic of the setup is shown in Fig. 4a, b. The system (details in “Methods”) only consists of a holographic metasurface and an OLED, enclosed inside a box. During measurement, the OLED was driven at 20 A/cm^2^ under DC operation. The holographic images were recorded using a charge-coupled device (CCD) camera. When the OLED was 5 cm away from the metasurface, the holographic image of the Dalmatian was observed (Fig. 4c). We note that due to the size of the projected image and the travel range of the translation stage, we only recorded the head of the Dalmatian image. While the main features of the image are clearly recognisable, compared with the image recorded with laser illumination, the image with OLED illumination was blurred, and most features on the right-hand side were missing. A bandpass filter was then inserted between the OLED and the metasurface to control the FWHM of the electroluminescence spectrum, without altering the distance between the OLED and the metasurface. As shown in Fig. 2b, the FWHM of the spectrum was reduced from 63 nm to 10 nm. The corresponding image quality significantly increased (Fig. 4d), with clear and sharp features over the whole image, thus successfully demonstrating our aim of combining an OLED and metasurface for the far-field projection of a designed holographic image. The minimum line feature generated by the OLED was measured to be approximately 300 µm. We also calculated the speckle contrast noise of the recorded image (supporting information) to be 0.23, which is much lower than reported for a hologram under laser illumination (0.81 for DPSS laser^28^). This further highlights the advantage of using OLED illumination.

**Fig. 5:**
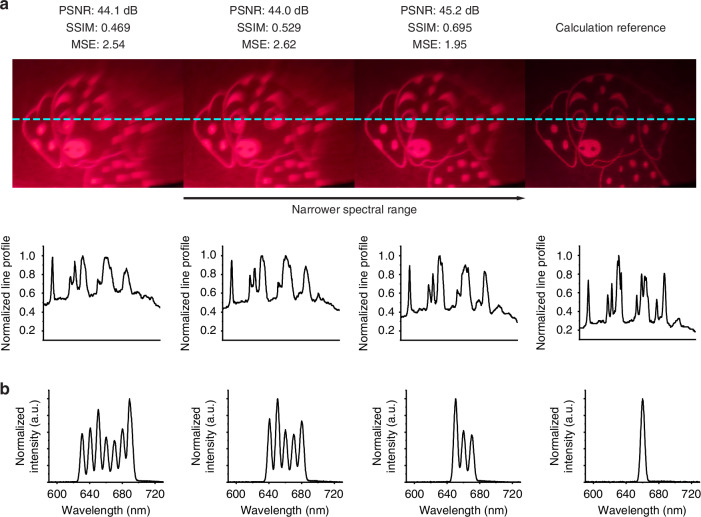
The effect of full width half maximum of illumination spectrum on image quality. **a** Recorded holographic image illuminated by SuperK laser (Centre wavelength 660 nm), cutline profile (cyan dashed line), calculated reconstructed accuracy, including peak signal-to-noise ratio (PSNR), structural similarity index (SSIM) and mean squared error (MSE). A narrower excitation spectral range was used from left to right. **b** Measured corresponding laser spectrum

To better understand how the low spatial coherence and a broad electroluminescence spectrum of the OLED affect the image quality, we first investigated the effect of spectral bandwidth. We used the same supercontinuum source used to generate the results of Fig. 3f, but tuned its coupled acousto-optic modulator to simulate a broadband light source. This approach has the advantage of retaining a high spatial coherence while examining the effect of the bandwidth of the spectrum on the quality of the holographic image. Different combinations of wavelengths were used to illuminate the metasurface. The centre wavelength was set at 660 nm, which was close to the peak of the OLED spectrum. The bandwidth of the spectrum was broadened by activating more wavelength channels on both sides of the centre wavelength with a step of 10 nm. Images recorded with different sets of laser wavelengths and the cutline profile (cyan dashed line) are shown in Fig. 5a, and the corresponding laser spectra are shown in Fig. 5b. For the image on the left panel, a spectral range of 60 nm (630 nm to 690 nm) was used. This spectral range is similar to the FWHM of the OLED EL spectrum. The projected holographic image was similar to the OLED illuminated holographic image, with the image quality decreasing towards the right part of the image. By narrowing the spectral range, an improved image quality was observed. Using the image recorded with the narrowest bandwidth (10 nm) as a reference, we calculated the peak signal-to-noise ratio (PSNR), structural similarity index (SSIM), and mean squared error (MSE) of the images recorded with broadband laser illumination (see supporting information). The quantitative analysis showed an improved reconstructed accuracy with a narrower spectral range.

We also examined the projected image under laser illumination with individual wavelengths and measured the distances between the image and zeroth-order diffraction peak (Fig. S12) which increased with longer illumination wavelengths, as expected. The image observed in Fig. 4c could therefore be considered as a superposition of multiple images under different wavelengths of illumination. We thus attribute the blurred image to the chromatic dispersion from the broad electroluminescence spectrum of the OLED.

We also find that the spatial coherence length at the metasurface influences image quality. Figure 2c shows that the spatial coherence length may be manipulated by varying the distance between the OLED and the metasurface, giving a spatial coherence length of 130 µm at 3 cm and 170 µm at 6 cm separation. Holographic images formed with the OLED at different distances are shown in Fig. 1. At distances from 3 to 6 cm, we were still able to observe the projected image. It is clear that the outline is sharper when the OLED is 6 cm away compared to 3 cm, showing how the spatial coherence length of the illuminating lights impacts the image sharpness. To compare the image quality, we quantitatively analysed the image quality at different distances and show the results in the supporting information. The quality of the images remained high even though the spatial coherence length was well below the length of a side of the metasurface (500 µm). We note that the minimum distance at which we could illuminate the metasurface was 3 cm, which results in a very compact holographic projector system.

## Discussion

Although OLEDs have lower output power density than lasers, which leads to a holographic image with lower brightness, OLEDs show advantages such as simplicity of fabrication, potential to be flexible and the feasibility of making large numbers of pixels of different colour side by side on the same substrate. All these advantages make OLEDs very suitable for future holographic display applications.

Whilst our proof of principle demonstration uses a bandpass filter to engineer the spectral width of the OLED, a thin film filter or polariton filter^21,31,32^ could be integrated with the OLED or the metasurface to make the system more compact. Our OLED illuminated holographic projector could be used in applications such as augmented reality / virtual reality headsets, human-computer interactions and portable holographic displays.

In terms of metasurface, our system could also work with another type of metasurface (e.g. all polymeric metasurfaces^33^). This offers significant potential for the mass production of these devices, facilitating their future deployment for image projection.

To summarise, we have demonstrated a compact OLED-illuminated metasurface system that can manipulate the OLED far-field emission and project holographic images. To enable the direct manipulation of OLED emission, we investigated the spatial coherence by using Young’s double slit experiment. The spatial coherence of the OLED was enhanced by extending the OLED and metasurface distances. Clear holographic images were obtained at an OLED metasurface distance as small as 3 cm. We also found that high spatial coherence and narrow FWHM of the emission spectrum are important to achieve high-quality images. We believe the findings in this work pave the way to a fully integrated and efficient self-illuminating holographic metasurface display.

## Materials and methods

### Fabrication of OLED

We fabricated the OLED by thermally evaporating materials in a vacuum chamber at a base pressure of 10^−7^ mbar (Angstrom Engineering Inc., Evo Vac) through custom-made shadow masks. The details of the fabrication of the OLED were described as the Red-OLED in the ref. ^34^. Onto pre-cleaned silicon substrates with 300 nm thick oxide layers, we first evaporated 300 nm thick aluminium as the anode, 3 nm thick MoO_3_ as hole injection layer, 70 nm 2,2′,7,7′-tetrakis(*N, N*′-di-*p*-methyl phenylamino)-9,9′-spirobifluorene (Spiro-TTB) doped with 2,2′- (perfluoronaphthalene-2,6-diylidene)dimalononitrile (F6-TCNNQ) (4 wt%) as hole transport layer, 10 nm *N, N*′-di(naphthalene-1-yl)-*N, N*′-diphenylbenzidine (NPB) as electron blocking layer, 4-(dicyanomethylene)-2-*tert*-butyl-6-(1,1,7,7-tetramethyljulolidin-4-yl-vinyl)-4*H*-pyran (DCJTB) at 1.5 vol% and Alq_3_ doped at 42 vol% in 3,9-di(naphthalen-2-yl)perylene and 3,10-di(naphthalen-2-yl) perylene mixture (DNP) with a thickness of 14 nm as emission layer, 10 nm tris(8-hydroxyquinolinato) aluminium (Alq_3_) as hole blocking layer, 40 nm caesium-doped 4,7-diphenyl-1,10-phenanthroline (Bphen) as electron transport layer, 30 nm silver layer as the cathode and a 50 nm thick NPB capping layer. After the evaporation, the OLEDs were encapsulated under a nitrogen atmosphere using custom-made glass lids with a cavity (Luminescence Technology Corp.), an epoxy glue (Norland Products Inc., Norland Optical Adhesive 68) and a moisture getter (Dynic Corporation, HD-071210T-50S).

### Meta-atoms Simulation

COMSOL Multiphysics was used to simulate the unit cell structure. The dispersion data for microscope glass (bk7), indium tin oxide (ITO), PMMA, and ZrO_2_ were all measured using an ellipsometer and are available in supporting information (Fig. S14). A linearly polarized plane wave (E-field along the x-axis) was used to excite the structure at normal incidence. It is noteworthy that the structure exhibits polarization independence under this illumination. Symmetrical boundary conditions were applied along the xz and yz planes, while open space boundaries were applied along the z-axis. The excitation wavelength was set to *λ* = 532 nm. It is important to note that the meta-atom illustrated in Fig. 3a, along with the qualitative results depicted in Fig. 3b, demonstrates versatility. These results can be replicated within the visible range by adjusting the lattice constant dimension. Detailed discussions about the metasurface design can be found in the supporting information.

### Fabrication of a holographic metasurface

The ZrO_2_ metasurface was fabricated on a microscope slide, which was initially cleaned in an ultrasonic bath using acetone and isopropanol for 5 min each. A 20 nm layer of ITO was subsequently deposited as a thin conductive layer via sputtering (Angstrom Engineering) to reduce electron backscattering effects. Following this, a 700 nm thick layer of PMMA A7 950 K (Micro Resist Technology) was spin-coated at 1650 rpm for 60 s and then baked for 5 min at 180 °C.

The meta-atoms pattern was defined using a 30 kV Raith eLine Plus Electron Beam Lithography (EBL) system, followed by a 1 min development process utilising a 1:1 mixture of isopropanol and distilled water. This development ratio was meticulously optimised to ensure complete development of all unit cells, regardless of size. Post-development, the sample was baked for an additional 30 min at 95 °C to enhance its thermal stability and mechanical strength. Finally, the ZrO_2_ layer was deposited using Atomic Layer Deposition at a rate of 1.79 Å per cycle at a temperature of 80 °C.

### OLED characterisation

We tested the current density-voltage-luminance characteristics of the OLED with a source measure unit (Keithley Instruments, Keithley 2400), a calibrated custom-made silicon photodiode module, and a multimeter (Keithley Instruments, Keithley 2000). We measured the emission area from an EL image of the operating OLED under a microscope (ECLIPSE LV100ND, Nikon) and found the active area is 5 × 10^−4^ cm^2^ (=124 × 404 µm^2^). We measured emission spectra with a calibrated fibre-coupled spectrograph (MS125, Oriel) equipped with a charge-coupled device (CCD) camera (DV420-BU, Andor) driving the OLED at 20 A/cm^2^

The spatial coherence of the OLED was characterised by Young’s double slit experiment. Briefly, interference patterns of the OLED at different distances from the double slits were recorded using a CCD camera (STF-8300M, SBIG). The width of the double slits was 40 µm and the centre-to-centre distance of the slits was 250 µm. The fringe visibility *V* of the interference pattern was calculated using the following equation:

$$V=\frac{{I}_{\max }-{I}_{\min }}{{I}_{\max }+{I}_{\min }}$$

where *I*_max_ is the central peak irradiance and *I*_min_ is the neighbouring valley irradiance. The spatial coherence length was then calculated based on a Gaussian–Schell model as follows:

$$V=\exp \left(-{\left|\vec{{\rho }_{1}}-\vec{{\rho }_{2}}\right|}^{2}/{L}_{c}^{2}\right)$$

where $\vec{{\rho }_{1}}$ and $\vec{{\rho }_{2}}$ are the vectors denoting two different positions on the plane perpendicular to the OLED propagation direction, and *L*_*c*_ is the spatial coherence length.

### Holographic metasurface characterisation

A broadband laser (SuperK, NKT photonics) coupled with an acousto-optic modulator tunable filter (SuperK SELECT, NKT photonics) was used to characterise the holographic metasurface. Up to 8 different wavelengths can be used simultaneously to illuminate the sample. The holographic image was projected on a screen at an angle of 30° with respect to the normal to the metasurface. The images were taken using a Samsung Galaxy S24 Ultra phone and an iPhone 15 camera in raw mode.

### OLED illuminated metasurface characterisation

The OLED on silicon substrate was mounted on a custom-made driver with a heat sink and cooling fan. Thermal conductive paste was applied between the OLED and the heat sink to promote heat dissipation during OLED operation. The OLED unit was then aligned and fixed close to the metasurface at the desired distance (3 to 6 cm). An optional bandpass filter was inserted between the OLED and the metasurface. A thin film aperture was stuck to the backside of the metasurface to block light from the OLED from being transmitted around the metasurface.

To record the holographic image, the OLED was driven at a constant voltage of 10 V. The holographic image was projected to a CCD camera (STF-8300M, SBIG) at an angle of 30°. A biconvex lens with a focal distance of 60 mm was used to collect and focus the image. The camera was mounted on a translation stage to take multiple images at different positions. All images were scaled to the same intensity range and then manually stitched to assemble the final holographic images.

## Supplementary information

Supporting information

## Data Availability

The research data underpinning this publication can be accessed at 10.17630/054a4b0d-4faa-4b2a-866f-8cb0982c0ff8^35^.
