# Supplementary material for: OLED illuminated metasurfaces for holographic image projection
Source: Light Sci Appl. 2025 Aug 27;14:294. doi: 10.1038/s41377-025-01912-z (PMC12391372; doi:10.1038/s41377-025-01912-z)
Supplement: Supplementary file 1 — Supporting information [file 41377_2025_1912_MOESM1_ESM.docx]

Supporting information for:

**OLED illuminated metasurfaces for holographic image projection**

Junyi Gong^1^, Mohammad Biabanifard^1^, Kou Yoshida^1^, Graham A. Turnbull^1, *^, Andrea Di Falco^1, *^, Ifor D.W. Samuel^1, *^

*^1^SUPA, School of Physics and Astronomy, University of St Andrews, North Haugh, St Andrews, Fife, KY16 9SS, United Kingdom*

**To whom correspondence should be addressed. E-mail: gat@st-andrews.ac.uk; adf10@st-andrews.ac.uk; idws@st-andrews.ac.uk*

**Table of content**

1. Design of OLED
2. Fabricated devices
3. Tolerance to imperfections of fabrication
4. Performance of reported metasurface
5. Additional characterisation of the metasurface
6. Speckle contrast noise
7. Reconstructed accuracy
8. Holographic images under laser illumination
9. Quality of holographic images under OLED illumination
10. Design of meta-atoms
11. Meta-atoms 16-level phase discretization
12. Diffraction efficiency
13. Metasurface design
14. **Design of OLED**


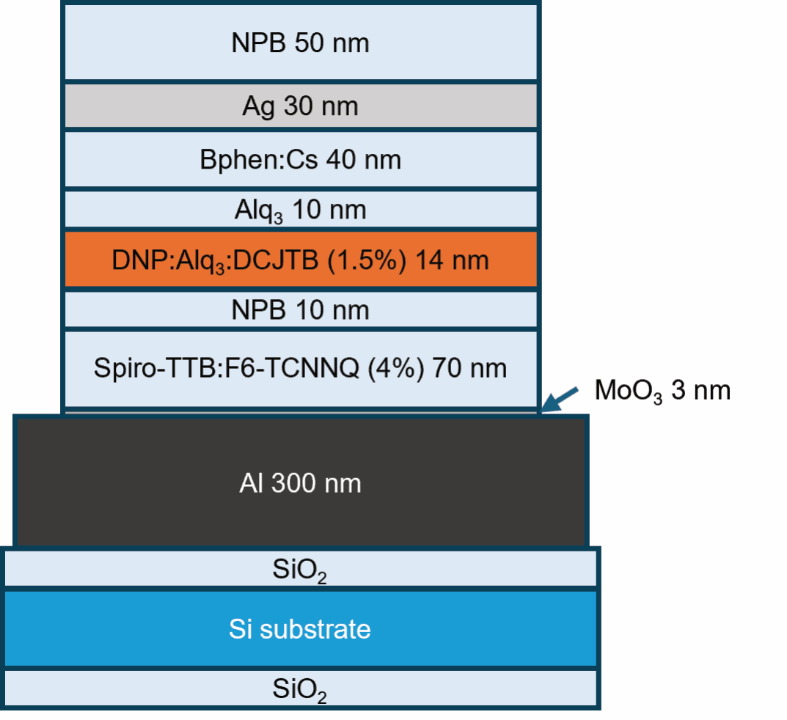


Figure S1 Schematic of the OLED device stack.

1. **Fabricated devices**

**
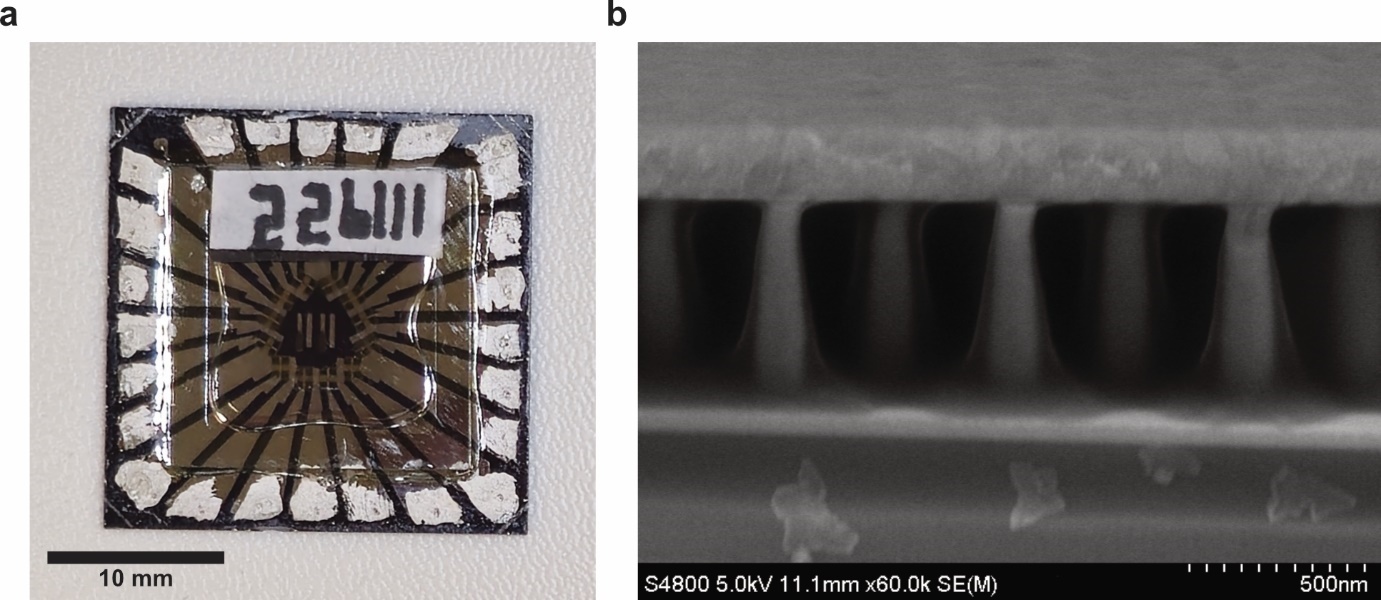
**

Figure S2 Images of the fabricated device. **a**, photo of the fabricated OLED sample. **b**, cross-section SEM image of the fabricated metasurface sample.

1. **Tolerance to imperfections of fabrication**

We have conducted multiple fabrication runs giving a yield of over 90%, during which we cleaved the metasurface (MS) samples at the midpoint and captured several scanning electron microscope (SEM) images to evaluate the MS structure and fabrication quality. Based on the consistent quality observed across different fabrication runs, we identified a deviation in the verticality of the PMMA pattern walls. This deviation stems from our 30 kV electron beam lithography system used to create patterns in a 700 nm-thick PMMA layer. The lack of perfectly straight walls impacts the targeted phase modulation, particularly for the larger pillars. To quantify this effect, we calculated the wall angle deviation for the most significant deviation case using larger pillars obtained from the SEM images in Fig. S3a and simulated the impact using COMSOL, as shown in Fig. S3b.


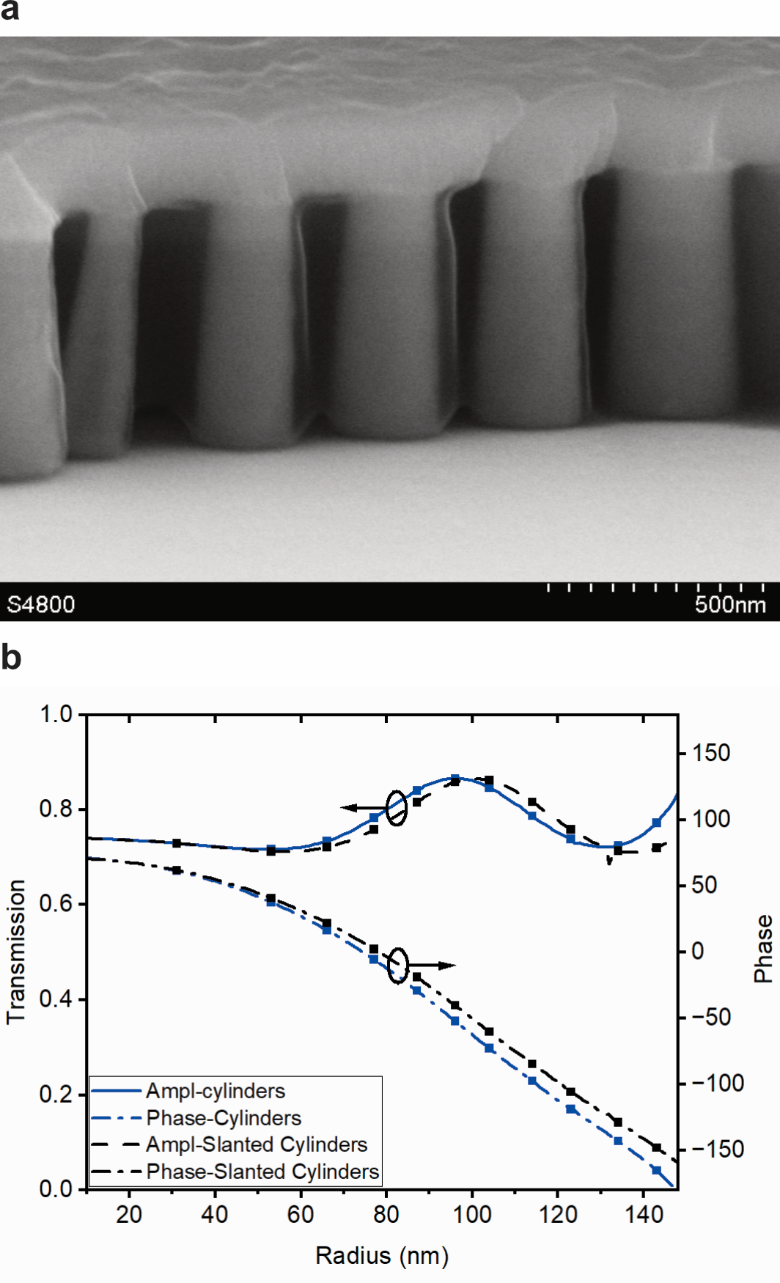


Figure S3 The effect of fabrication imperfections. **a**, SEM image of a fabricated meta-atoms for larger pillars, **b**, amplitude and phase modulation error created by fabrication imperfections. The filled squares indicate the fabricated meta-atoms.

The results indicate that the amplitude remains qualitatively unchanged, while the phase modulation error shows a deviation of less than 10% from the intended phase value. Since the slanted walls for most radii produce a simple shift of the phase, the effect on the quality of the image is very small. Accordingly, Fig. S3b demonstrates that the metasurface exhibits significant robustness to fabrication imperfections.

1. **Performance of reported metasurface**

**Table S1 Summary of the performance of reported metasurface.**

| Ref. No. | Year | Focusing efficiency | Diffraction efficiency |
| --- | --- | --- | --- |
| 1 | 2015 | Not relevant | 80% |
| 2 | 2015 | 80% | Not relevant |
| 3 | 2014 | Not relevant | 75% |
| 4 | 2017 | Not relevant | 43% |
| 5 | 2014 | Not relevant | 83% |
| 6 | 2017 | 12.44% | Not relevant |
| 7 | 2015 | Not relevant | 59.2% |
| 8 | 2016 | 90% | Not relevant |
| 9 | 2015 | 82% | Not relevant |
| 10 | 2013 | Not relevant | 18% |
| 11 | 2016 | Not relevant | 23.7% |
| 12 | 2024 | 47% | Not relevant |
| 13 | 2024 | Not relevant | 26% |
| 14 | 2024 | Not relevant | 67% |
| This work | 2024 | Not relevant | 27% |

1. **Additional characterisation of the metasurface**

Our metasurface is broadband, which can work with a wide range of wavelengths covering the entire visible light spectrum. To demonstrate theoretically the effect of polarization and wavelength variations, we utilised COMSOL to simulate the designed meta-atoms optimised for 532 nm, under excitation at 485 nm and 660 nm. The impact on phase and amplitude modulation is presented in Fig. S4.


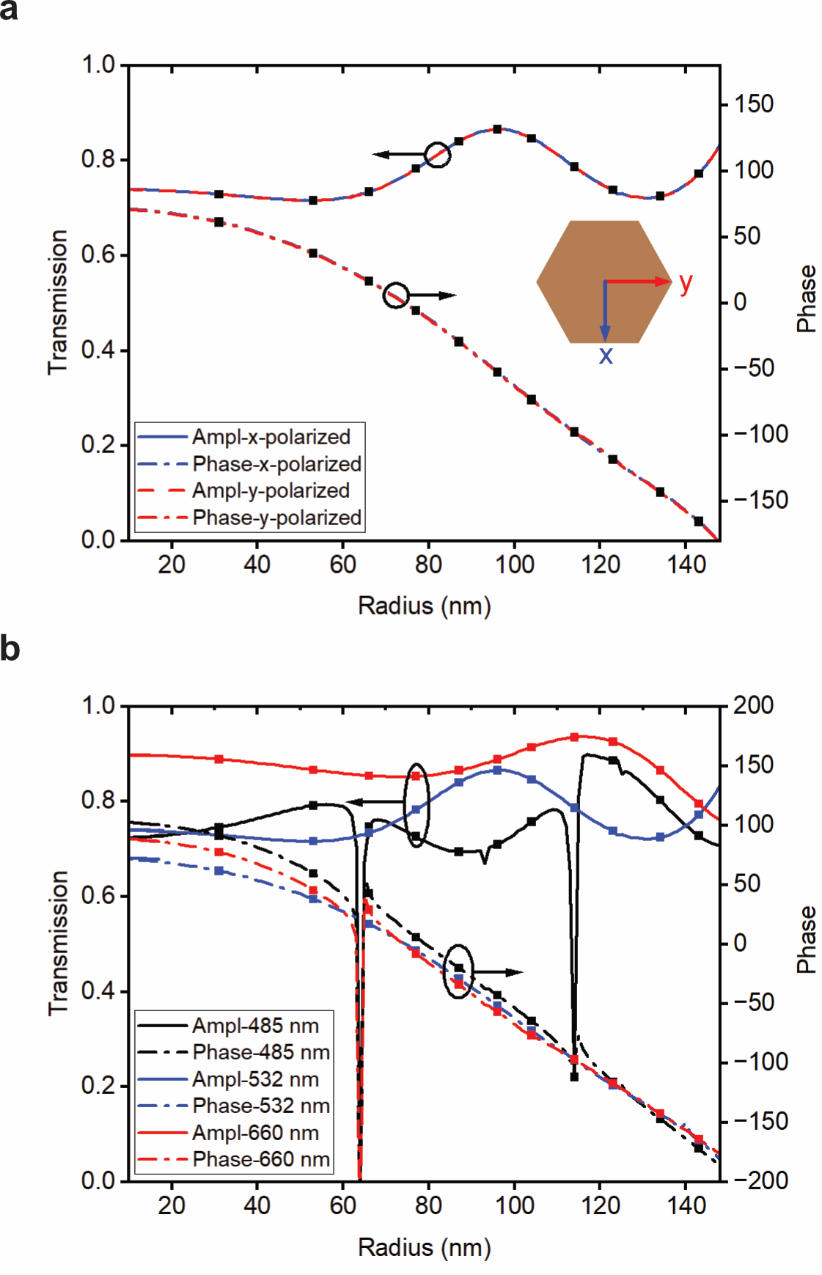


Figure S4 Excitation of the designed meta-atoms at normal incidence. a, showing x- and y- polarizations at 532 nm b, comparing 485 nm, 532 nm and 660 nm illumination for x-polarization.

We also characterised the metasurface under laser illumination with different polarizations, wavelengths, and illumination angles, as shown in Fig. S5, S6 and S7.


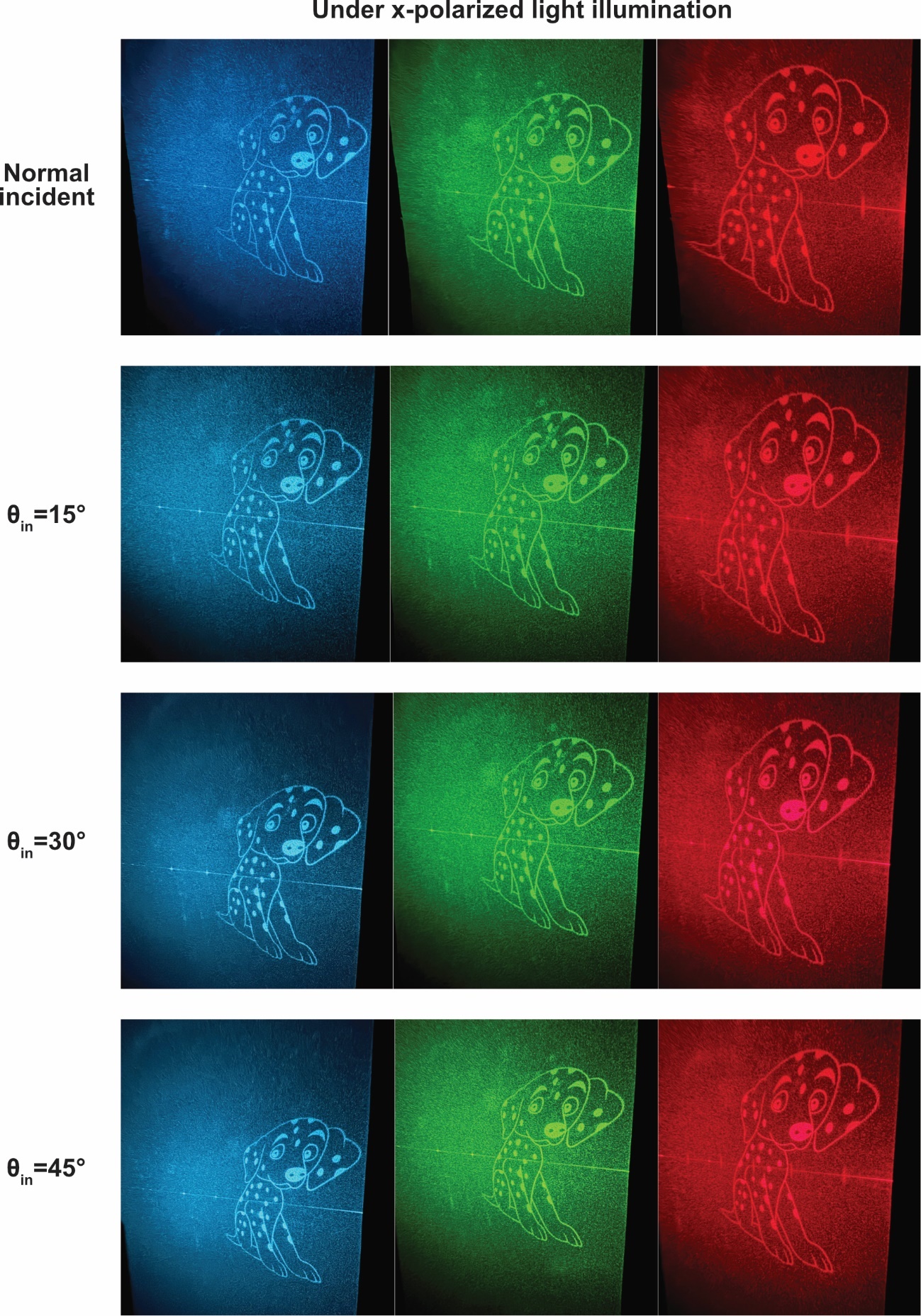


Figure S5 Recorded holographic images using x-polarized light under SuperK illumination at varying incident angles, with excitation wavelengths of 485 nm, 532 nm, and 660 nm.


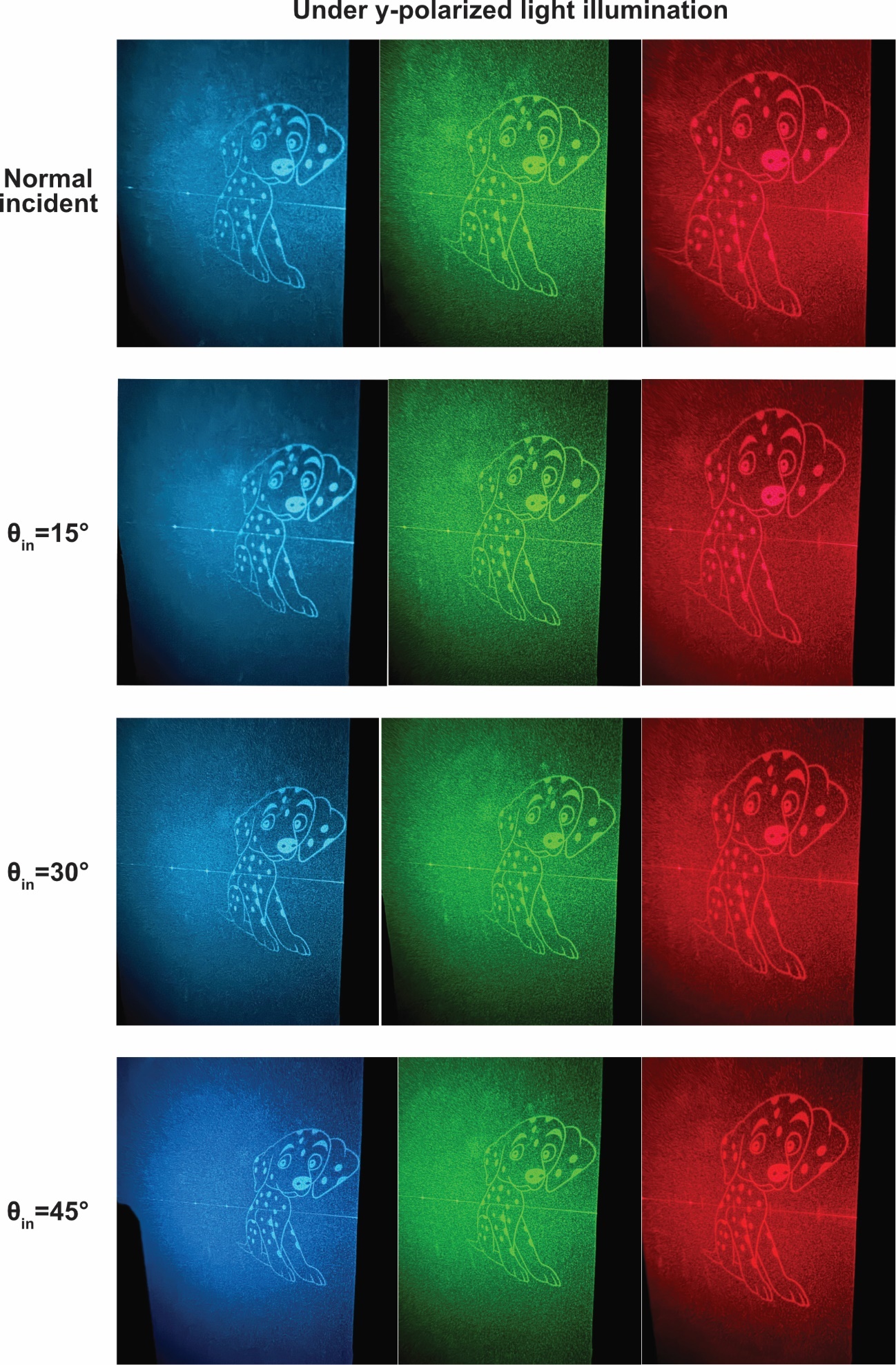


Figure S6 Recorded holographic images using y-polarized light under SuperK illumination at varying incident angles, with excitation wavelengths of 485 nm, 532 nm, and 660 nm.


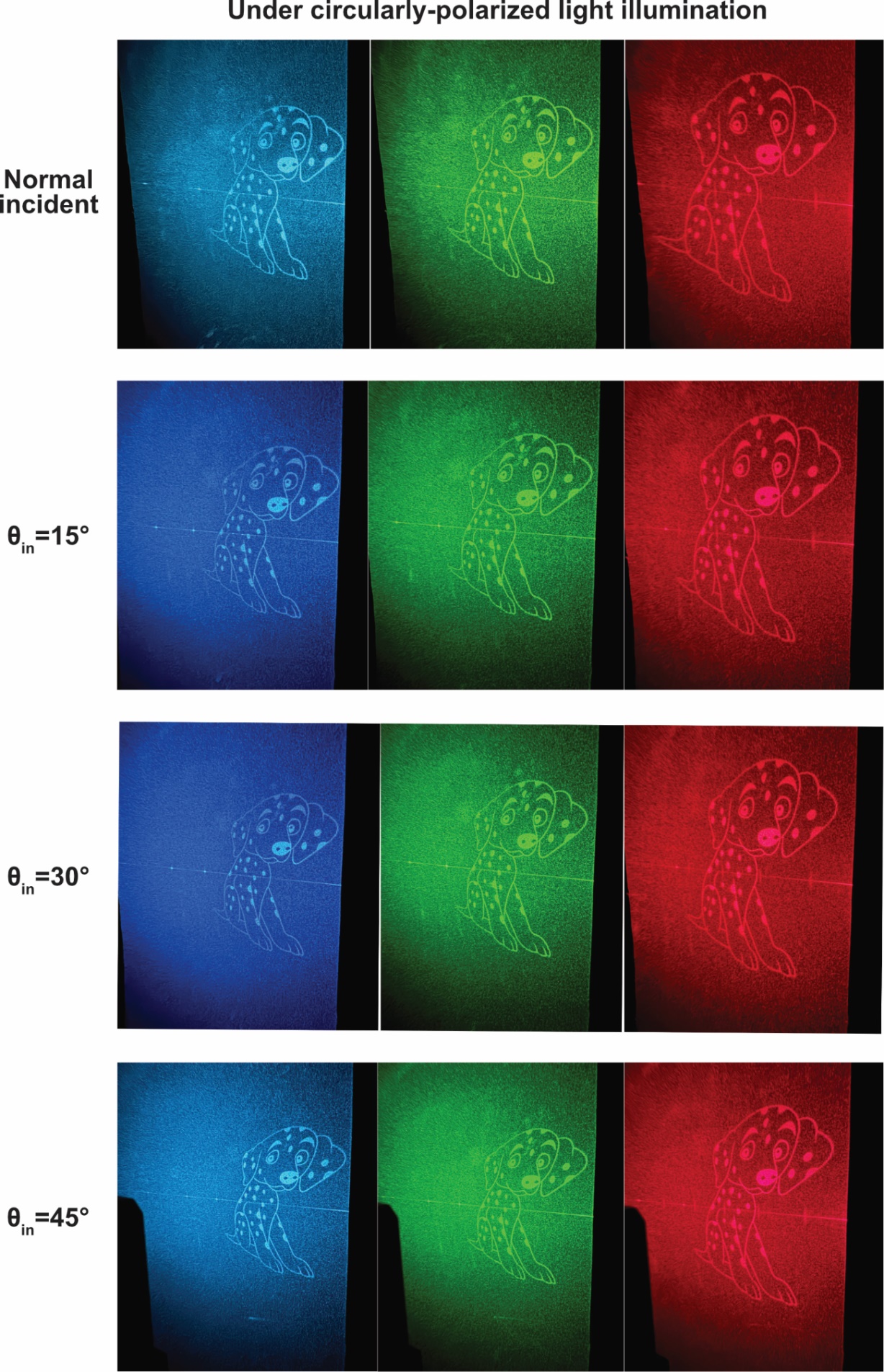


Figure S7 Recorded holographic images using circularly-polarized light under SuperK illumination at varying incident angles, with excitation wavelengths of 485 nm, 532 nm, and 660 nm.

It should be noted that in recording all the images for each set of polarization under different off-axis illumination, the laser beam, camera, and screen positions were fixed while the sample was tilted to the intended angle. This explains why the images appear skewed for higher illumination angles. Adjusting the angle of the screen would restore the shape of the image.

We further quantify the polarization effect by calculating the contrast-to-noise ratio (CNR) and signal-to-noise ratio (SNR) of the holographic image under laser illumination using the following equations. The region of interest for calculation is shown in Fig. S8.

$$CNR=\frac{\left| S_{A}-S_{B} \right|}{\sigma_{0}}$$

Where $S_{A}$ and $S_{B}$ are signal intensities for signal producing structures A and B in the Region Of Interest (ROI) and $\sigma_{0}$ is the standard deviation of the pure image noise.

$$SNR=\frac{\mu_{sig}}{\sigma_{sig}}$$

Where $\mu_{sig}$ is the average signal value and $\sigma_{sig}$ is the standard deviation.


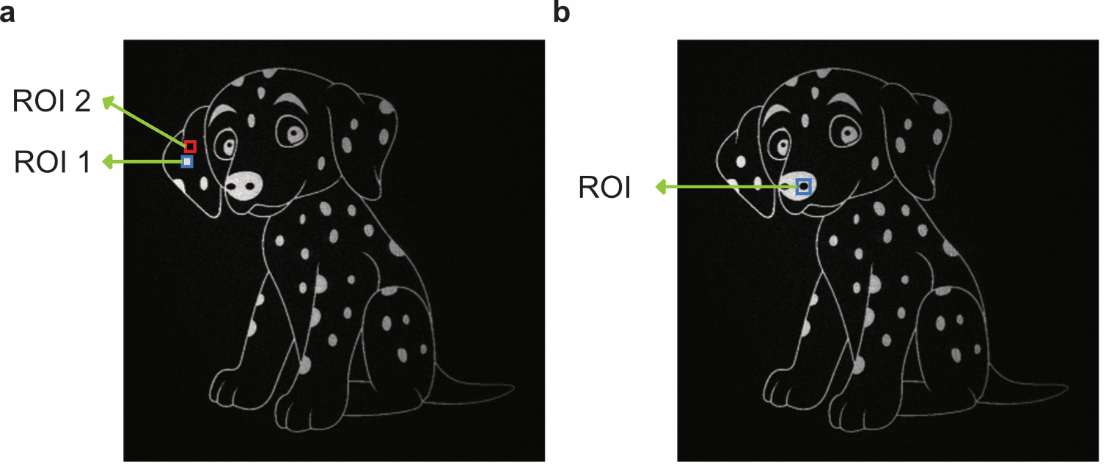


Figure S8 Region Of Interest for calculation of **a**, contrast-to-noise ratio and **b**, signal-to-noise ratio.

Based on the recorded image, we calculated the CNR under different illumination with different polarizations, different incident angles and at different wavelengths. The calculated values were plotted in Fig. S9. Under x-polarized light illumination, the CNR of the recorded images was affected by the incident angle, especially at 532 nm. A similar effect was observed for y-polarized light at 660 nm wavelength. For both polarizations, CNR decreased with longer wavelengths.


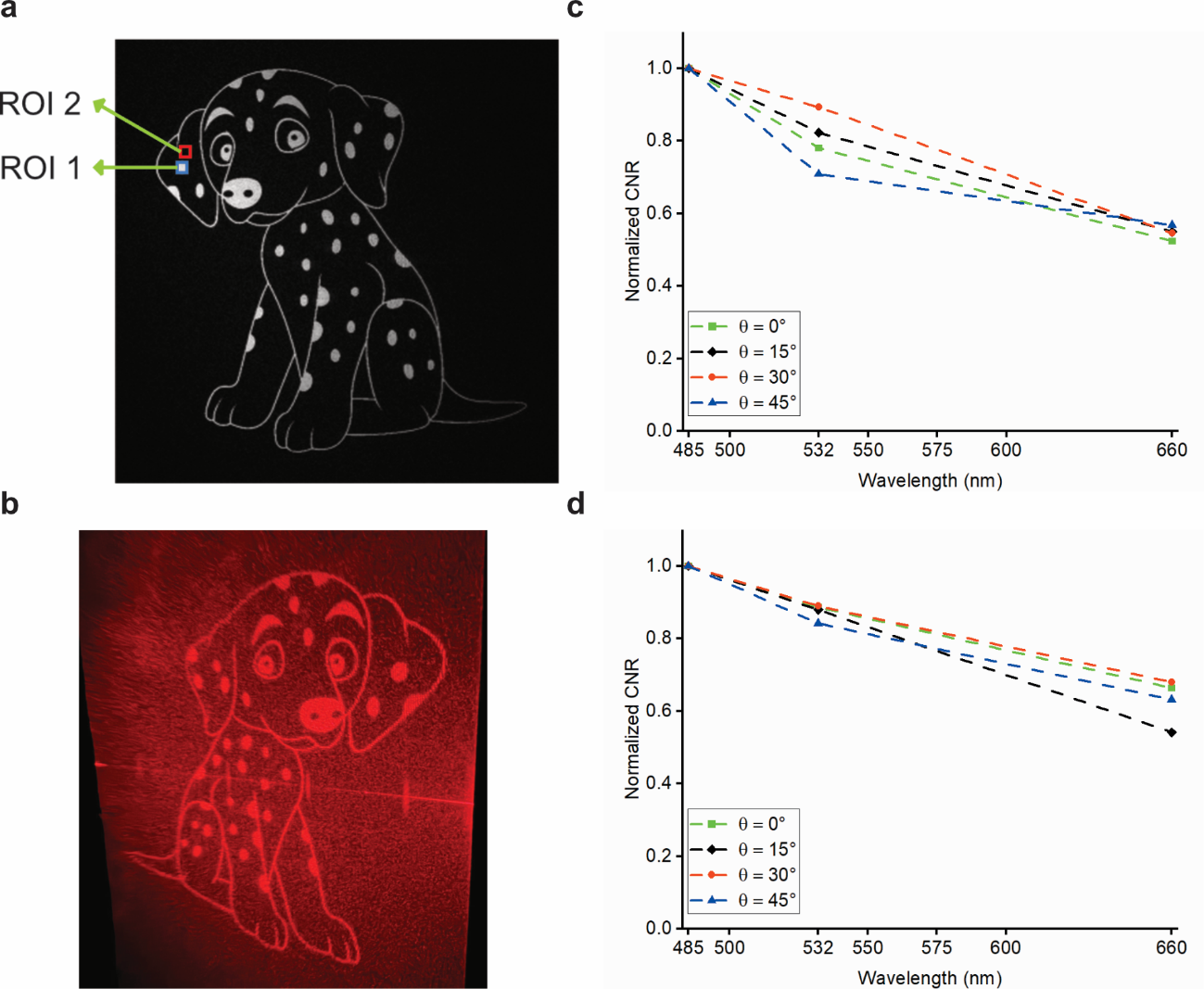


Figure S9 Calculated contrast-to-noise ratio of the recorded images under different polarizations, different incident angles, and different wavelengths. **a**, RS simulated image, **b**, recorded image at 660 nm, **c**, CNR for x-polarized light, and **d**, CNR for y-polarized light.

The SNR value was plotted in Fig. S10. Similar as CNR, SNR was affected by the incident angle and decreased at longer wavelengths. For both CNR and SNR, at a given wavelength, the impact of polarization and angle of incidence on image quality was very small. Hence, our experimental results clearly demonstrate that the designed metasurface exhibits polarization insensitivity and possesses a high tolerance to off-axis illumination.


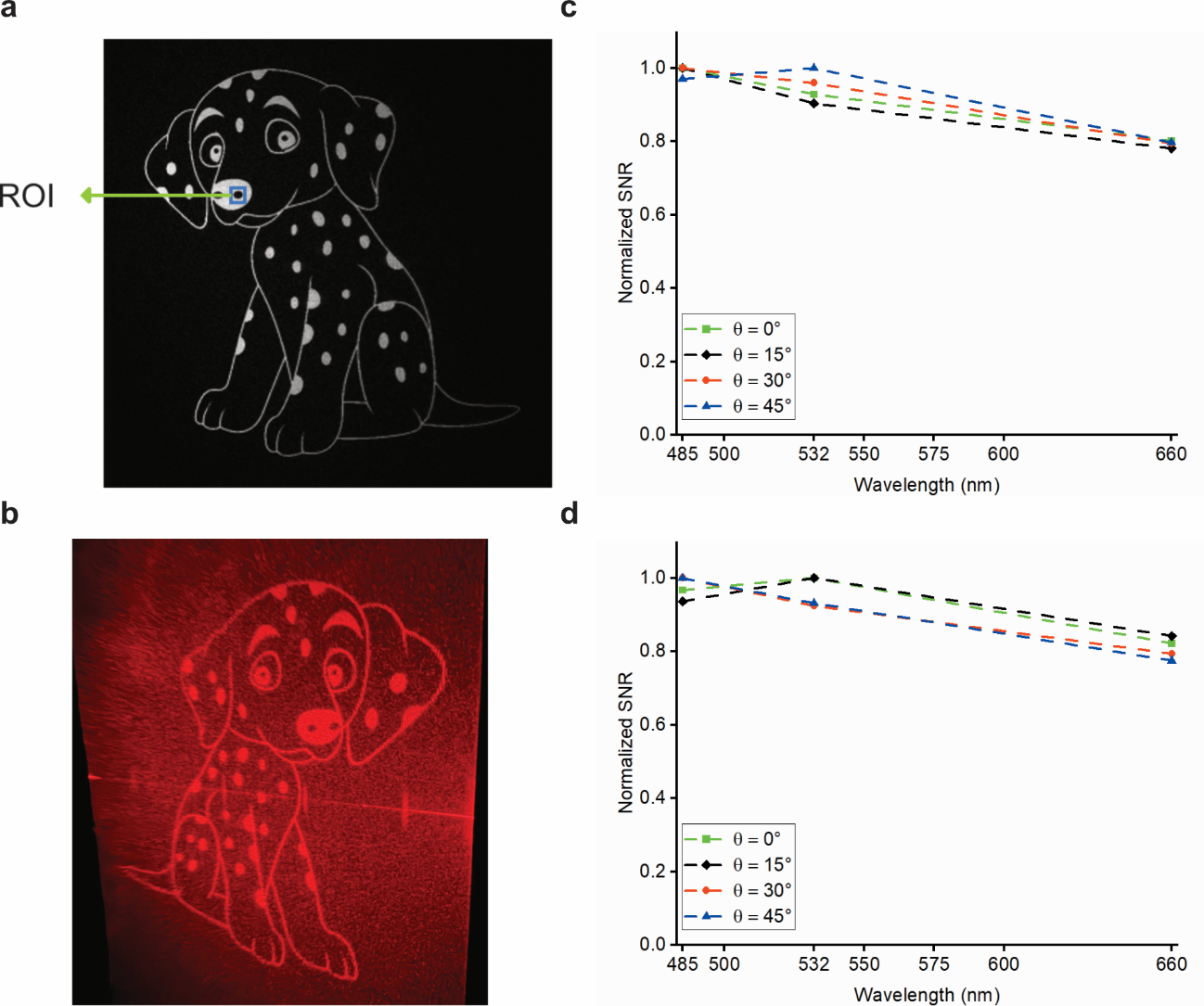


Figure S10 Calculated signal-to-noise ratio of the recorded images under different polarizations, different incident angles, and different wavelengths. **a**, RS simulated image, **b**, recorded image at 660 nm, **c**, SNR for x-polarized light, and **d**, SNR for y-polarized light.

1. **Speckle contrast noise**

We note that speckle noise is very rarely reported for holographic metasurfaces. To quantify the speckle noise in our work, we calculated the speckle contrast C of the projected image using the following equation^15^:

$$C=\frac{\sqrt{\frac{1}{MN}\sum_{i=1,j=1}^{M,N} {(I_{i,j}-\bar{I})}^{2}}}{\bar{I}}$$

Where *M* and *N* are the row and column values of the calculated area, $I_{i,j}$ is the intensity value of a pixel at a particular position and $\bar{I}$ is the average intensity of the calculated area.

We selected part of the nose region (Fig. S11) of the recorded holographic image under OLED illumination and calculated the speckle contrast C to be 0.23. This value is much lower than reported for a hologram under laser illumination (0.81 for DPSS laser^15^). Hence, the lower coherence of OLEDs is an advantage for reducing speckle.


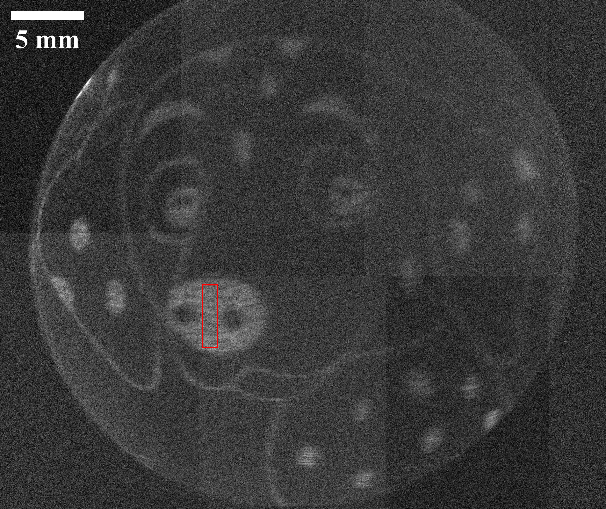


Figure S11 Recorded holographic image under OLED illumination with bandpass filter. The region which was used to calculate speckle contrast is highlighted by the red square.

1. **Reconstructed accuracy**

**Table S2 Reconstructed accuracy of recorded images under broadband light illumination.**

| Illumination bandwidth | PSNR | SSIM | MSE |
| --- | --- | --- | --- |
| [nm] | [dB] |  |  |
| 20 | 45.2 | 0.695 | 1.95 |
| 40 | 44.0 | 0.529 | 2.62 |
| 60 | 44.1 | 0.469 | 2.54 |

We calculated the peak signal-to-noise ratio (PSNR)^16^, structural similarity index (SSIM)^17^ and mean squared error (MSE)^16^ of the image recorded in Figure 5 (manuscript) and summarise the results in the above table.

$$MSE=\frac{1}{mn}\sum_{i=0}^{m-1} \sum_{j=0}^{n-1} \left[ I\left( i,j \right)-K\left( i,j \right) \right]^{2}$$

$$PSNR=10\cdot\log_{10} \left( \frac{{MAX}_{I}^{2}}{MSE} \right)$$

Where $I\left( i,j \right)$ and $K\left( i,j \right)$ are intensity and noisy approximation of a noise-free $m\times n$ monochrome image. ${MAX}_{I}$ is the maximum possible pixel value of the image. For example, for a pixel represented by 8 bits, this value is 255.

$$SSIM\left( x,y \right)=\frac{\left( 2\mu_{x}\mu_{y}+c_{1} \right)\left( 2\sigma_{xy}+c_{2} \right)}{\left( \mu_{x}^{2}+\mu_{y}^{2}+c_{1} \right)\left( \sigma_{x}^{2}+\sigma_{y}^{2}+c_{2} \right)}$$

Where $\mu_{x}$ and $\mu_{y}$ are the pixel sample mean of $x$ and $y$, $\sigma_{x}^{2}$, $\sigma_{y}^{2}$ and $\sigma_{xy}$ are the variance and covariance of $x$ and $y$. $c_{1}=\left( k_{1}L \right)^{2}$ and $c_{2}=\left( k_{2}L \right)^{2}$ are two variables to stabilise the division of with weak denominator. $L$ is the dynamic range of the pixel-values ($2^{bits per pixel}-1$). By default, $k_{1}=0.01$ and $k_{2}=0.03$.

1. **Holographic images under laser illumination**


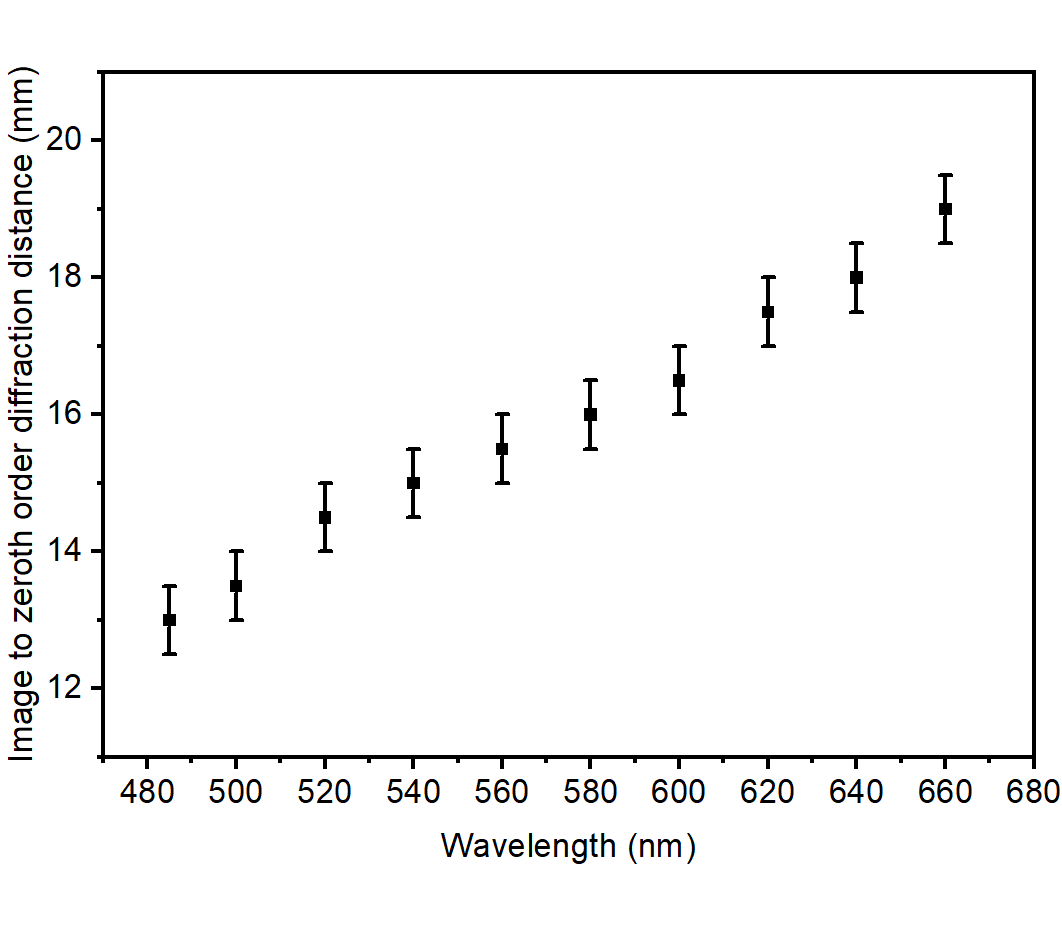


Figure S12 Image to zeroth order diffraction distance as a function of illumination wavelength.

1. **Quality of holographic images under OLED illumination**

To assess quantitatively how the quality of the reconstructed images depends on spatial coherence, we calculated the SSIM of the experimental images using the target image as reference. Since the dimension of the experimental images change with the distance between the MS and the camera, for each case we scaled the ROI of the target image to match the size of the experimental ones. The SSIM was calculated according to Ref [17]. Here, for estimating local statistics around each pixel, we used an isotropic Gaussian function with a standard deviation equal to 15 pixels. As expected, the SSIM increases with the degree of coherence of the source, where doubling the distance between OLED and MS from 3cm to 6cm leads to an increase of 19% in the SSIM. Fig. S13c shows the cutline profile of the experiment ROI images.


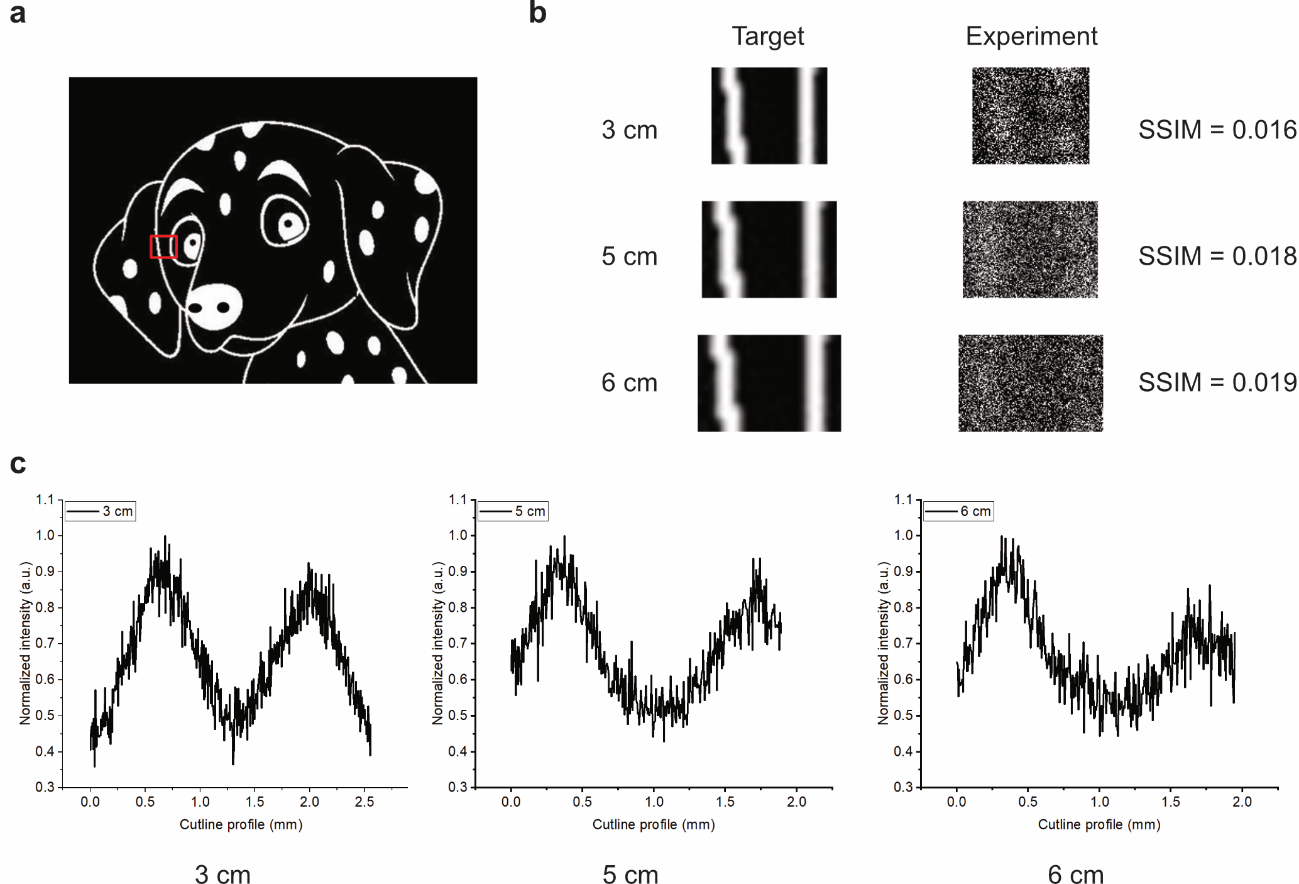


Figure S13 **a**, Zoomed-in view of the target image. The ROI used to calculate the SSIM is highlighted by the red box. **b**, ROI of the scaled target image and experimental cropped-up images for different distances between the OLED and the metasurface. **c**, Cutline profile of the experimental ROI images.

1. **Design of meta-atoms**


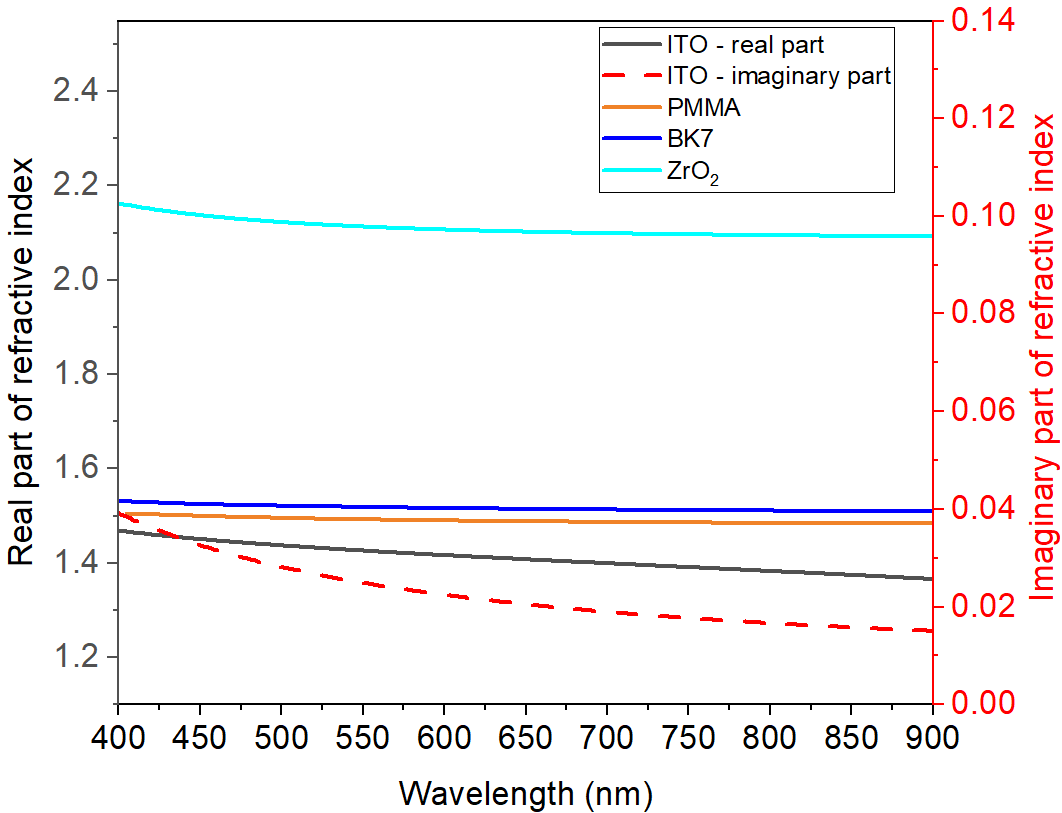


Figure S14 Refractive index of materials used in meta-atoms design.

1. **Meta-atoms 16-level phase discretization**

| **Table S3 Meta-atoms 16-level phase discretization** | | | | | | | | | |
| --- | --- | --- | --- | --- | --- | --- | --- | --- | --- |
|  | | | | | | | | | |
| **λ = 532 nm** | **Phase** | -46° | -24° | -2° | 20° | 42° | 64° | 86° | 108° |
|  | **Radius** | 114 nm | 104 nm | 96 nm | 87 nm | 77 nm | 66 nm | 53 nm | 31 nm |
|  | **Phase** | 5° | 27° | 50° | 72° | 95° | -112° | -90° | -68° |
|  | **Radius** | - | - | - | - | - | 143 nm | 134 nm | 123 nm |

1. **Diffraction efficiency**

**
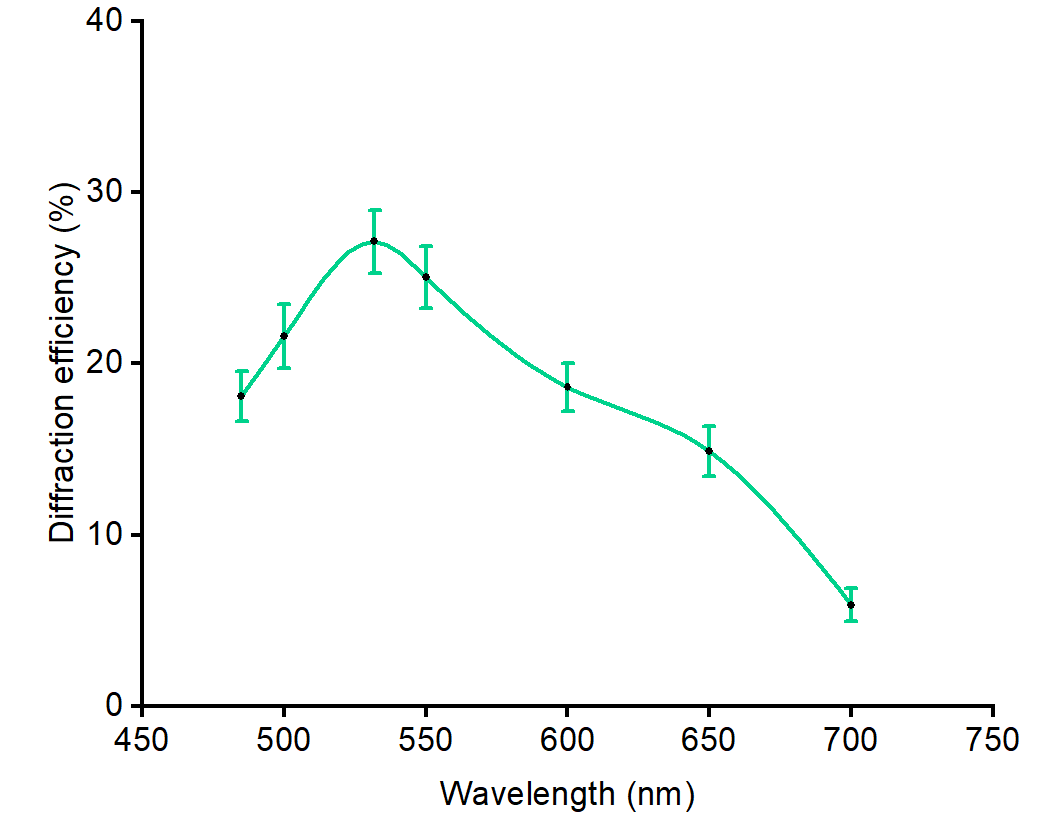
**

Figure S15 The diffraction efficiency of the ZrO₂ metasurface. The metasurface was optimised for a wavelength of 532 nm. The black dots represent the measured efficiency data, with error bars indicating the variation in efficiency across five identically designed metasurfaces, differing only in fabrication quality. The green line is provided as a visual guide.

1. **Metasurface design**

We utilised our customised Gerchberg-Saxton algorithm (GSA) to design the required phase profile at the interface, as described in references,^18,19^ to generate the holographic image of a Dalmatian dog. This algorithm generates the phase distribution of the holographic metasurface from the target image by incorporating parameters such as source intensity, source shape, wavelength, holographic distance, and hologram pitch. The GSA operates by propagating light between two spatially separated planes: the metasurface plane and the holographic image plane, as shown in Fig. S16.


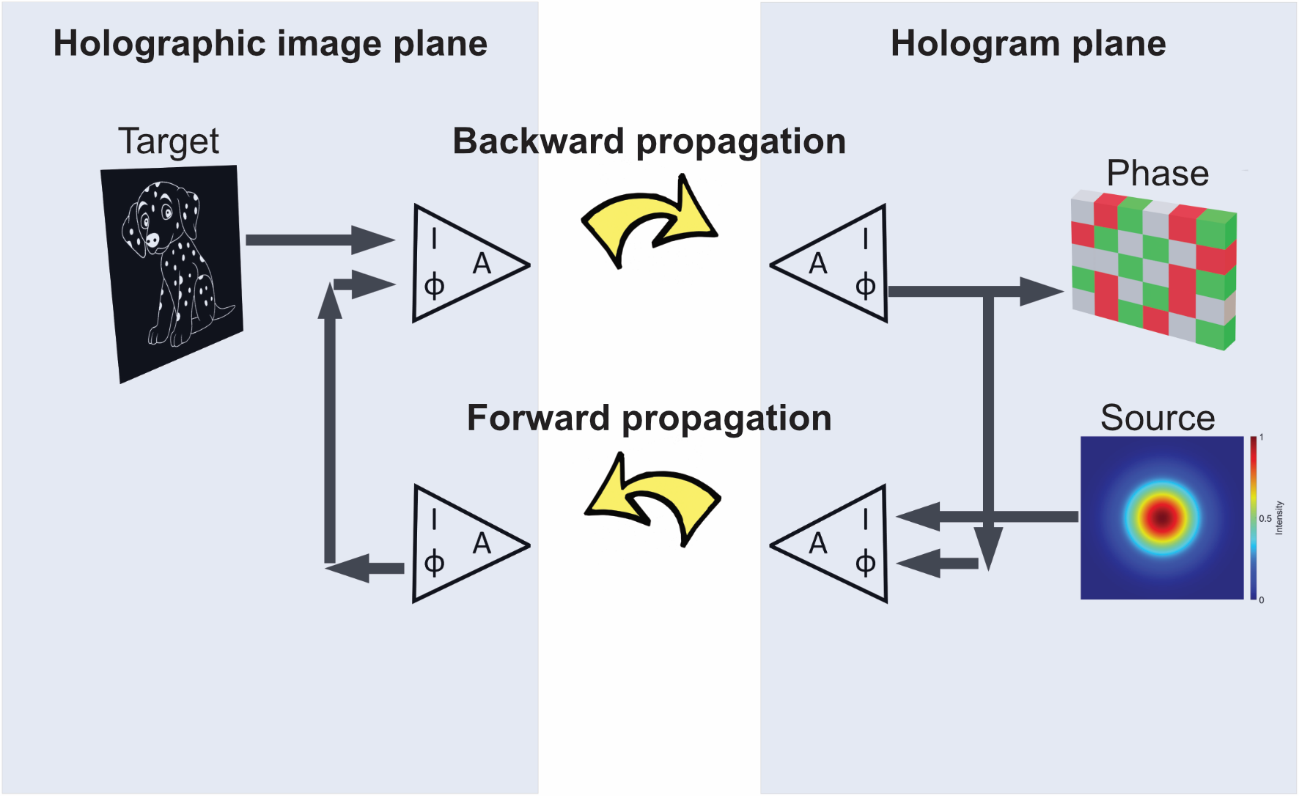


Figure S16 Graphical illustration of a simple GSA (A, Φ, and I represent amplitude, phase, and intensity, respectively).

As a phase retrieval algorithm, it iteratively converges to the correct hologram solution.^18^ Here, we describe it through seven steps: 1) set the holographic image as the target image. 2) Back-propagate the holographic image to the metasurface plane. 3) Replace the intensity achieved by propagation with the intensity of the source. 4) Propagate the updated complex field to the holographic image plane. 5) Replace the intensity with the intensity of the target image. 6) Calculate the least square differences of the absolute values between the holographic image and target image, and repeat steps 2-6 if this value is greater than a pre-defined value. 7) Return the hologram phase.

The propagation tool used to design the phase profile is critically important. The simplest approach involves using Fourier and inverse Fourier transforms to propagate and back-propagate light between two spatially separated planes. However, this basic method imposes design limitations, such as constraints on resolution and the minimum distance between the hologram and holographic plane, as the latter must reside in the far-field. More accurate results can be achieved through numerical calculations that work beyond the limit of validity of the Fast Fourier Transform methods. Here, to calculate the phase profile of the hologram we employed the Rayleigh-Sommerfeld (RS) method.^19^ Part of the calculated phase profile (100 by 100 pixel) is shown in Fig. S17.


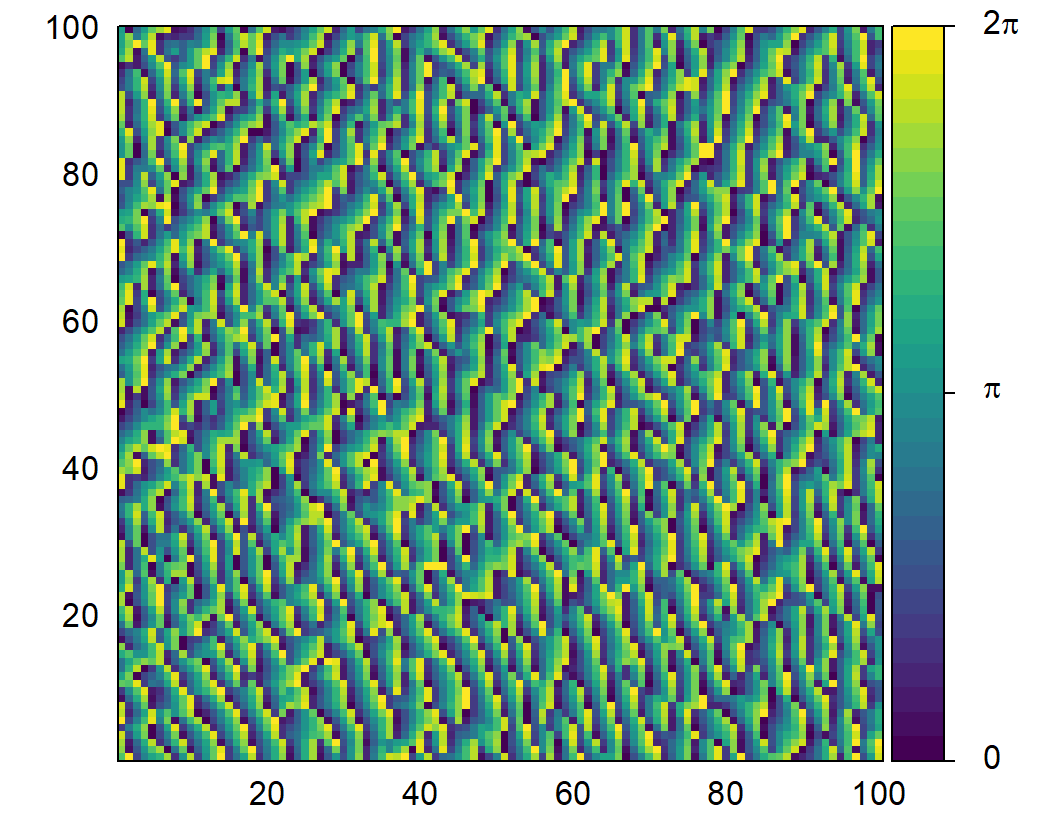


Figure S17 Zoom in (100 by 100 pixel) of the phase profile of the design hologram.

**References**

1 Zheng, G. *et al.* Metasurface holograms reaching 80% efficiency. *Nature Nanotechnology* **10**, 308-312 (2015).

2 Arbabi, A., Horie, Y., Bagheri, M. & Faraon, A. Dielectric metasurfaces for complete control of phase and polarization with subwavelength spatial resolution and high transmission. *Nature Nanotechnology* **10**, 937-943 (2015).

3 Lin, D., Fan, P., Hasman, E. & Brongersma, M. L. Dielectric gradient metasurface optical elements. *Science* **345**, 298-302 (2014).

4 Balthasar Mueller, J. P., Rubin, N. A., Devlin, R. C., Groever, B. & Capasso, F. Metasurface polarization optics: Independent phase control of arbitrary orthogonal states of polarization. *Physical Review Letters* **118**, 113901 (2017).

5 Yang, Y. *et al.* Dielectric meta-reflectarray for broadband linear polarization conversion and optical vortex generation. *Nano Letters* **14**, 1394-1399 (2014).

6 Wang, S. *et al.* Broadband achromatic optical metasurface devices. *Nature Communications* **8**, 187 (2017).

7 Wen, D. *et al.* Helicity multiplexed broadband metasurface holograms. *Nature Communications* **6**, 8241 (2015).

8 Khorasaninejad, M. *et al.* Polarization-insensitive metalenses at visible wavelengths. *Nano Letters* **16**, 7229-7234 (2016).

9 Arbabi, A., Horie, Y., Ball, A. J., Bagheri, M. & Faraon, A. Subwavelength-thick lenses with high numerical apertures and large efficiency based on high-contrast transmitarrays. *Nature Communications* **6**, 7069 (2015).

10 Chen, W. T. *et al.* High-efficiency broadband meta-hologram with polarization-controlled dual images. *Nano Letters* **14**, 225-230 (2014).

11 Li, X. *et al.* Multicolor 3d meta-holography by broadband plasmonic modulation. *Science Advances* **2**, e1601102 (2016).

12 Yao, J. *et al.* Nonlocal meta-lens with huygens' bound states in the continuum. *Nature Communications* **15**, 6543 (2024).

13 Wang, J. *et al.* Unlocking ultra-high holographic information capacity through nonorthogonal polarization multiplexing. *Nature Communications* **15**, 6284 (2024).

14 Biabanifard, M., Plaskocinski, T., Xiao, J. L. & Di Falco, A. Zro_2_ holographic metasurfaces for efficient optical trapping in the visible range. *Advanced Optical Materials* **12**, 2400248 (2024).

15 Deng, Y. & Chu, D. Coherence properties of different light sources and their effect on the image sharpness and speckle of holographic displays. *Scientific Reports* **7**, 5893 (2017).

16 Chervyakov, N., Lyakhov, P. & Nagornov, N. Analysis of the quantization noise in discrete wavelet transform filters for 3d medical imaging. *Applied Sciences* **10** (2020).

17 Wang, Z., Simoncelli, E. P. & Bovik, A. C. in *The Thrity-Seventh Asilomar Conference on Signals, Systems & Computers, 2003.* 1398-1402 Vol.1392.

18 Gerchberg, R. W. A practical algorithm for the determination of plane from image and diffraction pictures. *Optik* **35**, 237-246 (1972).

19 Burch, J. & Di Falco, A. Surface topology specific metasurface holograms. *ACS Photonics* **5**, 1762-1766 (2018).
